# Mental and Emotional Self-Help Technology Apps: Cross-Sectional Study of Theory, Technology, and Mental Health Behaviors

**DOI:** 10.2196/mental.7262

**Published:** 2017-10-17

**Authors:** Benjamin T Crookston, Joshua H West, P Cougar Hall, Kaitana Martinez Dahle, Thomas L Heaton, Robin N Beck, Chandni Muralidharan

**Affiliations:** ^1^ Department of Health Science Brigham Young University Provo, UT United States

**Keywords:** mental health, decision theory, mobile applications

## Abstract

**Background:**

Mental and emotional self-help apps have emerged as potential mental illness prevention and treatment tools. The health behavior theory mechanisms by which these apps influence mental health–related behavior change have not been thoroughly examined.

**Objective:**

The objective of this study was to examine the association between theoretical behavior change mechanisms and use of mental and emotional self-help apps and whether the use of such apps is associated with mental health behaviors.

**Methods:**

This study utilized a cross-sectional survey of 150 users of mental or emotional health apps in the past 6 months. Survey questions included theory-based items, app engagement and likeability items, and behavior change items. Stata version 14 was used to calculate all statistics. Descriptive statistics were calculated for each of the demographic, theory, engagement, and behavior variables. Multiple regression analysis was used to identify factors associated with reported changes in theory and separately for reported changes in actual behavior after controlling for potentially confounding variables.

**Results:**

Participants reported that app use increased their motivation, desire to set goals, confidence, control, and intentions to be mentally and emotionally healthy. Engagement (*P*<.001) was positively associated with the reported changes in theory items, whereas perceived behavior change was positively associated with theory (*P*<.001), engagement (*P*=.004), frequency of use of apps (*P*=.01), and income (*P*=.049).

**Conclusions:**

Participants reported that app use increased their motivation, desire to set goals, confidence, control, and intentions to be mentally and emotionally healthy. This increase in perceptions, beliefs, and attitudes surrounding their mental and emotional health was considerably associated with perceived change in behavior. There was a positive association between the level of engagement with the app and the impact on theory items. Future efforts should consider the value of impacting key theoretical constructs when designing mental and emotional health apps. As apps are evaluated and additional theory-based apps are created, cost-effective self-help apps may become common preventative and treatment tools in the mental health field.

## Introduction

Mental health is a state of well-being in which every individual realizes their own potential, can cope with the normal stresses of life, can work productively and fruitfully, and is able to make a contribution to the community [1]. Many Americans struggle with diagnosable mental illness such as anxiety and depression [2]. According to the National Institute of Mental Health, the prevalence of anxiety disorders among US adults is 18.1%, with 22.8% of these cases classified as *severe* mental illness [2]. Major depressive disorder affects more than 15 million American adults or approximately 6.7% of the US adult population [3]. A number of other mental illnesses, including panic disorder, phobias, bipolar disorder, schizophrenia, obsessive compulsive disorder, posttraumatic stress disorder, eating disorders, sleep disorders, substance abuse disorders, and attention deficit or hyperactive disorders also affect millions of Americans [4]. Unfortunately, many individuals with mental illnesses do not seek help. Among those that do, there are often pervasive delays in receiving care [5]. Some have turned to self-help approaches to address and manage mental health and wellness concerns.

Self-help products constitute a multibillion dollar industry [6]. Books, podcasts, and mobile phone apps are among the most popular tools. In 2014, there were 4.55 billion mobile phone users worldwide [7]. By 2016, there were 198.5 million people in the United States who owned mobile phones with functioning apps [8]. Mental and emotional self-help apps have been developed recently to meet the demands resulting from growing interest and acceptability [9]. In a survey examining the potential of mobile phone apps, 76% of respondents expressed that they were interested in using free mobile phone apps to maintain their own mental health [10]. Such apps may promote changes in care-seeking behavior [11], reduce barriers to accessing mental health treatment [12], and increase the perceived value in mental health care services [13]. For example, Mobilyze is an app designed to manage depressive symptoms. One study concluded that Mobilyze users were satisfied with the app and improved considerably on self-reported depressive symptoms and interview-reported measures of depressive symptoms. Users had decreased comorbid anxiety symptoms and were less likely to meet criteria for major depressive disorder diagnosis [14].

Mental and emotional self-help apps have the potential to play an important role in the future of mental health prevention and treatment; however, intervention evaluations suggest that such apps should integrate theory-based principles of health behavior to effectively accomplish the goal of improving mental and emotional health [15].

Health behavior theory informs the development and execution of interventions to achieve program objectives [16]. This is accomplished by addressing the mechanisms by which individuals make changes to their behavior. For example, the Health Belief Model (HBM) is used to explain and predict health behaviors by focusing on individuals’ perceptions of what is likely to happen to them if they choose not to change their behavior [4]. The emphasis of social cognitive theory (SCT) is to promote change through improvements in self-efficacy and a combination of environmental- and individual-level characteristics [17,18]. According to the theory of planned behavior (TPB), human behavior is guided by behavioral, normative, and control beliefs [19]. Behavioral beliefs are the beliefs that an individual has about the probable outcomes of a behavior and the appraisals of the results. Normative beliefs are the expectancies of others and incentive to conform with these expectancies. Control beliefs are the attitudes about the presence of facilitating or impeding factors that may affect the functioning of the behavior and the influence of these aspects [20].

Although mental and emotional health self-help apps are widely used, the health behavior theory mechanisms by which these apps influence mental health-related behavior change have not been thoroughly examined [21]. Although a limited number of studies have explored the extent to which apps are founded on the principles of health behavior theory, these studies have not measured whether theoretical constructs are associated both with the use of apps and with health behavior change. The purpose of this study was to examine the association between theory-based behavior change mechanisms and use of mental and emotional self-help apps and whether the frequent use of and engagement with such apps were positively associated with perceived behavior change. We hypothesized that theoretical behavior change constructs, including knowledge, attitudes, beliefs, perceptions, self-efficacy, and intentions to behave, are related to app use and to perceived behavior change.

## Methods

### Design

This study utilized a cross-sectional survey directed to users of mental or emotional health apps in the past 6 months. The survey included questions about demographics, theory-informed items, as well as likeability and respondents’ engagement with the apps.

### Sample

A total of 207 individuals initiated the survey, with 171 meeting the inclusion criteria. Potential respondents were informed that the purpose of the study was to understand more about individuals’ use of technology to improve their health, and in particular, to learn more about the use of self-help apps that can be used to track mental and emotional health status. Some popular examples of these types of apps include Happify and MindShift. To qualify for the study, participants were required to live in the United States, be at least 18 years of age or older, and have used a mental or emotional self-help app in the previous 6 months. Surveys were only provided in English. Of the 171 participants who met the inclusion criteria, 150 participants answered all survey questions and were included in the final study sample.

### Procedure

An electronic Qualtrics survey was distributed through Amazon Mechanical Turk (MTurk). A pilot test involving 15 individuals was conducted before distribution of the survey to the main sample. Following the pilot test, changes were made to the survey questions and flow using feedback provided by pilot participants. The final survey link was uploaded to MTurk and participants were initially paid US $1 for completing the survey. A total of 52 surveys had been completed after 1 week of data collection. The survey was relaunched with a compensation of US $2, and additional 119 respondents participated in the survey. The participants’ MTurk IDs were required to initiate the survey and were used to verify each survey to ensure the authenticity of the participant and to prevent duplications. Duplicates were not allowed to initiate the survey.

### Measurement

Theory-based behavior change and likeability items were adapted from the previous studies of health apps. The adaptation made for this study included changes to make the items more applicable for app users’ own personal experience with apps [22,23]. Demographic items in the survey included age, gender, race, ethnicity, level of education, state of residence, and combined annual household income. Using health behavior theories, Likert-type survey questions were developed to examine theory-based mechanisms of change, engagement with and likeability of the use of apps in the past 6 months, and changes in their actual behavior. Theory-based items included the following: “belief that poor mental and emotional health leads to bad health,” “belief that being mentally and emotionally healthy can prevent bad health,” “belief that diseases related to poor mental and emotional health are harmful,” “belief that mental and emotional health is important in preventing bad health,” “motivation to be mentally and emotionally healthy,” “ability to be mentally and emotionally healthy,” “confidence that I can be mentally and emotionally healthy,” “control over my mental/emotional health,” “intentions to be mentally and emotionally healthy,” “attitudes about the importance of being mentally and emotionally healthy,” “belief that people important to me want me to be mentally and emotionally healthy,” “perception that many other people are mentally and emotionally healthy,” “knowledge of the diseases/disorders that are caused by poor mental and emotional health,” “knowledge of ways in which I can be mentally and emotionally healthy,” “awareness of the benefits of being mentally and emotionally healthy,” “desire to be mentally and emotionally healthy,” “social support I have received for being mentally/emotionally healthy,” “positive feedback I have received for being mentally/emotionally healthy,” “desire to set goals to be mentally/emotionally healthy,” and “ability to achieve my mental/emotional health goals.” A composite variable was constructed summing the Likert score for all theory-related constructs to provide a broad-based theory variable. Provided the complexities relating to mental and emotional health, a practical approach to behavior change may involve a combination of distinct constructs and elements from each theory, effectively forming a polytheoretical approach. The Cronbach alpha coefficient for this composite variable was .947. This variable was not normally distributed and a square root transformation was used to normalize it.

Engagement with and likeability of the app were assessed using the following items: “the app(s) was helpful,” “the app(s) was easy to use,” “I enjoyed using the app(s),” “I liked the app(s),” and “I would recommend the app(s) to others.” A composite engagement variable was computed to estimate the total level of engagement and likeability of the reported use of apps in the 6 months before the study period. The Cronbach alpha coefficient for this composite variable was .921.

Perceived behavior change items included the following: “actual goal setting to be mentally /emotionally healthy,” “frequency of practicing mental/emotional management techniques,” “intensity to which I practice mental/emotional management techniques,” and “consistency in using mental/emotional health management techniques.” A composite perceived behavior change variable was computed by summing the responses to the behavior change questions. The Cronbach alpha coefficient for this composite variable was .900.

### Analysis

Stata version 14 was used to calculate all statistics. Descriptive statistics were calculated for each of the demographic, theory, engagement, and behavior variables. Multiple regression analysis was used to identify factors associated with reported changes in theory and separately for reported changes in actual behavior after controlling for potentially confounding variables, including app engagement, price of each app, frequency of use, gender, age, and annual income. Assumptions of independence were tested, and tolerance levels were within acceptable ranges.

## Results

Of the 150 participants, 57.3% were female, 78.7% were white, and 39.3% had a 4-year college degree (see Table 1).

Most participants agreed (44.0%) or strongly agreed (45.3%) that the mental or emotional self-help apps increased their motivation to be mentally and emotionally healthy (Table 2). Approximately half (48.7%) of the respondents agreed and 40.7% strongly agreed that the app increased their confidence that they can be mentally and emotionally healthy.

Mental and emotional self-help apps were perceived to increase the control over mental and emotional health (44.7% of respondents agreed and 38.7% strongly agreed), intentions to be mentally and emotionally healthy (38.0% agreed and 50.7% strongly agreed), attitudes about the importance of being mentally and emotionally healthy (46.0% agreed and 42.0% strongly agreed), desire to set goals to be mentally and emotionally healthy (46.0% agreed and 47.3% strongly agreed), and ability to achieve mental and emotional health goals (52.7% agreed and 36.0% strongly agreed). Participants reported varying levels of disagreement or neutrality with the following statements: the app(s) increased my belief that people important to me want me to be mentally and emotionally healthy (2.0% strongly disagree, 8.0% disagree, and 25.3% neutral); the app(s) increased my perception that many other people are mentally and emotionally healthy (1.3% strongly disagree, 15.3% disagree, and 26.0% neutral); the app(s) increased my knowledge of the diseases/disorders that are caused by poor mental health (1.3% strongly disagreed, 14.0% disagreed, and 17.3% neutral); the app(s) increased the social support I have received for being mentally/emotionally healthy (4.0% strongly disagreed, 18.0% disagreed, and 18.0% neutral); and the apps(s) increased the positive feedback I have received for being mentally/emotionally healthy (3.3% strongly disagreed, 13.3% strongly disagreed, and 14.7% neutral). The behavior item response distribution is depicted in Table 3.

**Table 1:** Summary of participant demographics.

Demographics		Frequency (N=150)
		n (%)
**Age, years**		
	18-25	13 (8.7)
	26-34	74 (49.3)
	35-54	55 (36.7)
	55-64	8 (5.3)
	65 or over	0 (0.0)
**Race or ethnicity**		
	Asian	11 (7.3)
	Black/African American	21 (14.0)
	White	118 (78.7)
**Gender**		
	Male	64 (42.7)
	Female	86 (57.3)
**Education**		
	Less than high school	2 (1.3)
	Diploma or GED^a^	16 (10.7)
	Some college	48 (32.0)
	2-year degree	17 (11.3)
	4-year degree	59 (39.3)
	Master’s degree	6 (4.0)
	Professional degree (Medical Doctor and Juris Doctor)	2 (1.3)
**Household income**^b^		
	Less than 30,000	38 (25.3)
	30,000-39,999	28 (18.7)
	40,000-49,999	17 (11.3)
	50,000-59,999	15 (10.0)
	60,000-69,999	17 (11.3)
	70,000-79,999	11 (7.3)
	80,000-89,999	6 (4.0)
	90,000-99,999	4 (2.7)
	100,000 or more	14 (9.3)

^a^GED: General Educational Development.

^b^All values are in 2016 US dollars.

**Table 2:** Summary of participant responses to theory questions.

Question^a^	Response (N=150)				
	Strongly disagree n (%)	Disagree n (%)	Neutral n (%)	Agree n (%)	Strongly agree n (%)
Increased my belief that poor mental and emotional health leads to bad health (SCT^b^ and TPB^c^)	4 (2.7)	13 (8.7)	20 (13.3)	80 (53.3)	33 (22.0)
Increased my belief that being mentally and emotionally healthy can prevent bad health (SCT and TPB)	2 (1.3)	6 (4.0)	20 (13.3)	74 (49.3)	48 (32.0)
Increased my belief that diseases related to poor mental and emotional health are harmful (SCT and TPB)	2 (1.3)	10 (6.7)	25 (16.7)	69 (46.0)	44 (29.3)
Increased my belief that mental and emotional health is important in preventing bad health (SCT and TPB)	2 (1.3)	8 (5.3)	15 (10.0)	71 (47.3)	54 (36.0)
Increased my motivation to be mentally and emotionally healthy (SCT)	2 (1.3)	3 (2.0)	11 (7.3)	66 (44.0)	68 (45.3)
Increased my ability to be mentally and emotionally healthy (SCT)	3 (2.0)	5 (3.3)	16 (10.7)	71 (47.3)	55 (36.7)
Increased my confidence that I can be mentally and emotionally healthy (SCT)	0 (0.0)	3 (2.0)	13 (8.7)	73 (48.7)	61 (40.7)
Increased my control over my mental/emotional health (TPB)	1 (0.7)	6 (4.0)	18 (12.0)	67 (44.7)	58 (38.7)
Increased my intentions to be mentally and emotionally healthy (TPB)	2 (1.3)	2 (1.3)	13 (8.7)	57 (38.0)	76 (50.7)
Increased my attitudes about the importance of being mentally and emotionally healthy (TPB)	0 (0.0)	1 (0.7)	17 (11.3)	69 (46.0)	63 (42.0)
Increased my belief that people important to me want me to be mentally and emotionally healthy (TPB)	3 (2.0)	12 (8.0)	38 (25.3)	51 (34.0)	46 (30.7)
Increased my perception that many other people are mentally and emotionally healthy (TPB)	2 (1.3)	23 (15.3)	39 (26.0)	48 (32.0)	38 (25.3)
Increased my knowledge of the diseases/disorders that are caused by poor mental and emotional health (SCT)	2 (1.3)	21 (14.0)	26 (17.3)	63 (42.0)	38 (25.3)
Increased my knowledge of ways in which I can be mentally and emotionally healthy (SCT)	0 (0.0)	2 (1.3)	10 (6.7)	79 (52.7)	59 (39.3)
Increased my awareness of the benefits of being mentally and emotionally healthy (HBM^d^)	0 (0.0)	4 (2.7)	13 (8.7)	71 (47.3)	62 (41.3)
Increased my desire to be mentally and emotionally healthy (TPB)	0 (0.0)	3 (2.0)	12 (8.0)	67 (44.7)	68 (45.3)
Increased the social support I have received for being mentally/emotionally healthy (SCT)	6 (4.0)	27 (18.0)	27 (18.0)	60 (40.0)	30 (20.0)
Increased the positive feedback I have received for being mentally/emotionally healthy (SCT)	5 (3.3)	20 (13.3)	22 (14.7)	71 (47.3)	32 (21.3)
Increased my desire to set goals to be mentally/emotionally healthy (SCT)	0 (0.0)	1 (0.7)	9 (6.0)	69 (46.0)	71 (47.3)
Increased my ability to achieve my mental/emotional health goals (SCT)	0 (0.0)	4 (2.7)	13 (8.7)	79 (52.7)	54 (36.0)

^a^All theory questions in the survey were preceded by the following statement: now think about the mental and emotional health app(s) that you have used in the past 6 months. Using the app(s) has...

^b^SCT: social cognitive theory.

^c^TPB: theory of planned behavior.

^d^HBM: health belief model.

**Table 3:** Summary of participant responses to behavior change questions.

Question^a^	Response (N=150)				
	Strongly disagree n (%)	Disagree n (%)	Neutral n (%)	Agree n (%)	Strongly agree n (%)
Increased my actual goal setting to be mentally/emotionally healthy	0 (0.0)	2 (1.3)	12 (8.0)	79 (52.7)	57 (38.0)
Increased my frequency of practicing mental/emotional management techniques	0 (0.0)	1 (0.7)	8 (5.3)	78 (52.0)	63 (42.0)
Increased the intensity to which I practice mental/emotional management techniques	0 (0.0)	1 (0.7)	20 (13.3)	81 (54.0)	40 (32.0)
Increased my consistency in using mental/emotional health management techniques	0 (0.0)	1 (0.7)	10 (6.7)	79 (52.7)	60 (40.0)

^a^All theory questions in the survey were preceded by the following statement: now think about the mental and emotional health app(s) that you have used in the past 6 months. Using the app(s) has...

The major proportion of participants agreed or strongly agreed that the app increased their actual goal setting to be mentally and emotionally healthy (52.7% agreed and 38.0% strongly agreed), their frequency of practicing mental and emotional management techniques (52.0% agreed and 42.0% strongly agreed), and their consistency in using mental and emotional health management techniques (52.7% agreed and 40.0% strongly agreed). Most participants reported that the app was helpful (40.0% agreed and 56.0% strongly agreed) and easy to use (40.0% agreed and 58.0% strongly agreed; Table 4).

In multivariate regression analyses, engagement with the app(s) (*P*<.001) was positively associated with the reported changes in the theory items after controlling for other variables in the model (Table 5). In the multivariate regression model with perceived behavior change as the dependent variable (Table 6), theory was positively associated (*P*<.001) as were engagement (*P*=.004), frequency of use of the app (*P*=.01), and income (*P*=.049) after controlling for other variables in the model.

**Table 4:** Summary of participant responses to engagement questions.

Question^a^	Response (N=150)				
	Strongly disagree n (%)	Disagree n (%)	Neutral n (%)	Agree n (%)	Strongly agree n (%)
The app was helpful.	0 (0.0)	1 (0.7)	5 (3.3)	60 (40.0)	84 (56.0)
The app was easy to use.	0 (0.0)	0 (0.0)	3 (2.0)	60 (40.0)	87 (58.0)
I enjoyed using the app.	0 (0.0)	3 (2.0)	12 (8.0)	50 (33.3)	85 (56.7)
I liked the app.	0 (0.0)	1 (0.7)	8 (5.3)	55 (36.7)	86 (57.3)
I would recommend the app to others.	0 (0.0)	2 (1.3)	15 (10.0)	53 (35.3)	80 (53.3)

^a^All engagement questions in the survey were preceded by the following statement: considering the mental and emotional health app(s) that you have used in the past 6 months...

**Table 5:** Regression to predict theory.

Variable	Standardized coefficient	Standard error	*P*>| *t* |	95% CI
App engagement	0.87	0.12	.001	0.63-1.11
Price of app	0.04	0.17	.81	−0.29 to 0.37
Frequency of app use	0.15	0.16	.36	−0.17 to 0.48
Female	1.00	0.15	.50	−0.19 to 0.39
Age	−0.06	0.10	.55	−0.25 to 0.13
Income	−0.03	0.03	.27	−0.08 to 0.02
_cons^a^	0.95	0.43	.03	0.11-1.80

^a^_cons: constant term.

**Table 6:** Regression to predict behavior change.

Variable	Standardized coefficient	Standard error	*P*>| *t* |	95% CI
Theory	0.27	0.04	0.001	0.20-0.35
App engagement	0.2	0.07	0.004	0.07-0.33
Price of app	0.07	0.08	0.39	−0.09 to 0.23
Frequency of app use	0.2	0.08	0.01	0.04-0.35
Female	−0.12	0.07	0.09	−0.25 to 0.02
Age	0.05	0.05	0.25	−0.04 to 0.15
Income	0.03	0.01	0.049	0.00-0.05
_cons^a^	0.69	0.05	0.001	0.28-1.09

^a^_cons: constant term.

## Discussion

### Principal Findings

The purpose of this study was to examine the extent to which using mental and emotional health apps may be positively associated with key theory-based constructs, which are believed to be associated with changes in behavior. Findings indicated that the mechanisms of health behavior theory were associated with both app use and perceived behavior change. These results suggest that mobile phone apps, which represent a nontraditional, low-cost approach to behavior change, may be a worthwhile tool for addressing mental health in individuals. The key theoretical constructs comprising the composite theory variables in this study were self-efficacy and behavioral intent. Participants reported increases in their motivation, desire to set goals, confidence, control, and intentions to be mentally and emotionally healthy from app use. These findings highlight the importance of increasing an individual’s self-efficacy when addressing mental and emotional health. Provided the high rates of mental and emotional health challenges together with the time and resource challenges traditionally required to address these challenges, the impact of apps on self-efficacy is encouraging. Respondents were largely favorable in their assessment of the apps’ impact, but some subtle differences emerged. For example, respondents almost universally said that using mental and emotional health apps increased their motivation, confidence, intentions, and attitudes. This is consistent with the findings from other studies showing theory integration into health apps, particularly for apps related to diet and physical activity [22,24]. When comparing constructs, social norms, social support, and feedback were all less impacted by the use of the app.

There are at least two paradigms that can be used to interpret these differences. As it relates to devices and behavior change, Fogg [25] introduced the idea of a functional triad. The triad is a framework that delineates the role of devices in the human-device interaction. According to the triad, devices can be tools, mediums, or social actors. The PRECEDE–PROCEED model is often used to conceptualize change determinants along three dimensions, predisposing, enabling, or reinforcing [24]. Mental and emotional health apps may involve one, two, or all three dimensions. Tools that are akin to predisposing factors increase the user’s capability (eg, knowledge and self-efficacy). Mediums that are similar to enabling factors facilitate an authentic experience for users (eg, assist in tracking). Finally, social actors, akin to reinforcing factors, assist the user in establishing and strengthening relationships (eg, social support and feedback). For example, mental and emotional health apps become tools or predisposing factors when used to diffuse information. Similarly, these apps serve as mediums or enabling factors when used by an individual to collect data regarding one’s personal behavior. Apps can be considered social actors or reinforcing factors because they allow users to interact with social support networks or resources. It might be that mental and emotional health apps are currently functioning mostly as tools or predisposing factors, which is the least involved utility of these apps, and perhaps one reason for respondents agreeing that these tools or predisposing constructs were most impacted by using a mental or emotional health app. Social support and feedback are examples of social actor or reinforcing dimensions that were measured in this study. Respondents reported less agreement with respect to the use of a mental or emotional health apps having an impact on these, which relates to findings from other research showing that health and fitness apps are not equipped to address these dimensions [24]. These patterns might also relate to the level of difficulty in integrating theory into the apps of this nature. Building an app to provide users with information to influence knowledge and attitudes may simply be easier than building an app that provides feedback and facilitates social support.

As a secondary purpose of this study, there was a positive association between the level of engagement with the app and the impact on theory items. This finding is even more important, given the positive association between reporting an impact on the mechanisms of theory and reporting a change in actual behavior. This finding provides support for other studies that are focusing on theory’s potential for changing diet and physical activity behaviors but for which no connection with behavior was established [22,23]. This may also provide general support for a more systematic attempt by developers to influence constructs that are believed to lead to health behavior change. Although this challenge has been noted previously [23], findings from this study may renew efforts to promote such an effort. Provided that mental and emotional health apps are anchored to theoretical constructs known to improve health behaviors and behavioral intentions generally, this medium has great value for individuals suffering from mental and emotional distress.

### Comparison With Prior Work

Recent and emerging research has helped to demonstrate that health-related apps can facilitate behavior changes. One study found that DBT Coach, a software app for a mobile phone, decreased users’ depression and general distress by providing coaching against maladaptive behavior and improving adaptive coping behaviors [21]. Another study reported that users of the Mobilyze app showed considerable improvement in self-reported depressive symptoms and interview measures of depressive symptoms. Mobilyze users also had decreased comorbid anxiety symptoms and became less likely to meet criteria for major depressive disorder diagnosis [14]. Additional mental and emotional self-help apps, including Get Happy Program [26], CopeSmart [27], Mobile Type, and Mobile Stress Management [9], have also shown significant potential for improving mental and emotional health. This study helps to move this body of research forward by identifying at least some of the mechanisms by which these changes occur. Findings from this study may lead to an increase in the commitment to address health behavior theory constructs to influence mental and emotional health outcomes.

### Errors and Limitations

The study findings should be interpreted in the context of its potential errors and limitations. Survey items were not divided or grouped together according to specific theories or health behaviors until the analysis phase of the study. Better planning may have resulted in more questions to evaluate additional mechanisms by which the apps impact behavior. Consequently, this study only explored mechanisms from two health behavior theories—SCT and TPB. Additionally, the study only included four questions to evaluate perceived behavior change from app use. Initially, forced responses on the survey questions were not all functional, so only 150 of the 171 participants who qualified for the survey completed all questions. This error resulted in a smaller sample size than desired. Furthermore, participants were originally compensated US $1 on MTurk for completing the survey, but the survey had to be relaunched at US $2 because not enough individuals completed the survey.

Several study limitations should be noted. One limitation is that the sample size was only 150 participants. A larger sample size may allow for more reliable data and greater population generalizability. Furthermore, survey method designs are inherently at risk of bias and inaccuracy as participants may not respond truthfully. Additionally, the study did not present a description of which apps may qualify as a mental or emotional self-help app. Consequently, there was a range of app classification types that participants subjectively determined as mental or emotional app. For example, participants may have used a meditation, prayer, faith-based scripture, medication adherence, mood tracker, stress management, or positive affirmation app. One survey question asked the participants which mental or emotional self-help apps they used in the past 6 months, but we did not research each survey response to verify whether their response included a real app available for download on mobile phones. Another limitation is the inherent demographic bias from using MTurk to gather survey responses. MTurk workers are a relatively homogenous group of individuals with a majority being white, middle-aged, and socioeconomically disadvantaged. The lack of demographic variability limits the generalizability of the data to the larger American population.

### Conclusions

This study examined the theoretical mechanisms by which mental and emotional self-help apps are associated with behavior. Participants reported that app use increased their motivation, desire to set goals, confidence, control, and intentions to be mentally and emotionally healthy. This increase in perceptions, beliefs, and attitudes surrounding their mental and emotional health was considerably associated with perceived change in behavior. These findings highlight the importance of increasing an individual’s self-efficacy when addressing mental and emotional health. Furthermore, there was a positive association between the level of engagement with the apps and the impact on theory items. Understanding how these self-help apps promote behavior change informs app producers and health providers to more effectively change the behavior and health of their clients. Future research should study which classification types of mental and emotional self-help apps (eg, meditation, prayer, faith-based scripture, medication adherence, mood tracker, stress management, or positive affirmation) are most effective in improving health outcomes and promoting mental wellness. Further research should use other health behavior theories apart from the two theories explored in this paper (SCT and TPB) to further explore the mechanisms by which behavior change occurs. To improve the validity of this study, future research may use objective indicators instead of perceived behavior change to more directly study behavior change. Specific apps should be researched to determine the ones that are most effective in reducing mental illness and improving mental and emotional wellness. Future efforts should consider the value of impacting key theoretical constructs when designing mental and emotional health apps. As this study revealed that social support and feedback were all less impacted by the use of the apps, future apps should be designed to impact social support and feedback as additional mechanisms to change behavior. As apps are evaluated and additional theory-based apps are created, cost-effective self-help apps may become common preventative and treatment tools in the mental health field.

Understanding whether these self-help apps are associated with health behaviors will inform app producers and health providers to more effectively address health behaviors of clients.

## Abbreviations

GED: General Educational Development
HBM: Health Belief Model
MTurk: Mechanical Turk
SCT: social cognitive theory
TPB: theory of planned behavior

## References

[1] (2014). WHO.

[2] (2014). NIMH.

[3] ADAA (2016). ADAA.

[4] Lennon JL (2005). The use of the health belief model in dengue health education. Dengue Bulletin.

[5] Demyttenaere K, Bruffaerts R, Posada-Villa J, Gasquet I, Kovess V, Lepine JP, Angermeyer MC, Bernert S, de Girolamo G, Morosini P, Polidori G, Kikkawa T, Kawakami N, Ono Y, Takeshima T, Uda H, Karam EG, Fayyad JA, Karam AN, Mneimneh ZN, Medina-Mora ME, Borges G, Lara C, de Graaf R, Ormel J, Gureje O, Shen Y, Huang Y, Zhang M, Alonso J, Haro JM, Vilagut G, Bromet EJ, Gluzman S, Webb C, Kessler RC, Merikangas KR, Anthony JC, Von Korff MR, Wang PS, Brugha TS, Aguilar-Gaxiola S, Lee S, Heeringa S, Pennell BE, Zaslavsky AM, Ustun TB, Chatterji S, WHO World Mental Health Survey Consortium (2004). Prevalence, severity, and unmet need for treatment of mental disorders in the World Health Organization World Mental Health Surveys. JAMA.

[6] Valiunas A (2010). The science of self-help. The New Atlantis.

[7] Zhang X (2017). Exploring the patterns and determinants of the global mobile divide. Telematics and Informatics.

[8] (2016). ComScore.

[9] Donker T, Petrie K, Proudfoot J, Clarke J, Birch MR, Christensen H (2013). Smartphones for smarter delivery of mental health programs: a systematic review. J Med Internet Res.

[10] Proudfoot J, Parker G, Hadzi Pavlovic D, Manicavasagar V, Adler E, Whitton A (2010). Community attitudes to the appropriation of mobile phones for monitoring and managing depression, anxiety, and stress. J Med Internet Res.

[11] Wendel S (2013). Designing for Behavior Change: Applying Psychology and Behavioral Economics.

[12] Watts SE, Andrews G (2014). Internet access is NOT restricted globally to high income countries: so why are evidenced based prevention and treatment programs for mental disorders so rare?. Asian J Psychiatr.

[13] Simon GE, Ludman EJ (2009). It's time for disruptive innovation in psychotherapy. Lancet.

[14] Burns MN, Begale M, Duffecy J, Gergle D, Karr CJ, Giangrande E, Mohr DC (2011). Harnessing context sensing to develop a mobile intervention for depression. J Med Internet Res.

[15] Bakker D, Kazantzis N, Rickwood D, Rickard N (2016). Mental health smartphone apps: review and evidence-based recommendations for future developments. JMIR Ment Health.

[16] Kok G, Schaalma H, Ruiter RA, van Empelen P, Brug J (2004). Intervention mapping: protocol for applying health psychology theory to prevention programmes. J Health Psychol.

[17] Bandura A (1997). Self-efficacy: The Exercise of Control.

[18] Bandura A (2005). The primacy of self-regulation in health promotion. Appl Psychol.

[19] Ajzen I, Kuhl J, Beckmann J (1985). From Intentions to Actions: A Theory of Planned Behavior. Action Control: From Cognition to Behavior.

[20] Ajzen I People.UMass.

[21] Rizvi SL, Dimeff LA, Skutch J, Carroll D, Linehan MM (2011). A pilot study of the DBT coach: an interactive mobile phone application for individuals with borderline personality disorder and substance use disorder. Behav Ther.

[22] West JH, Cougar Hall P, Arredondo V, Berrett B, Guerra B, Farrell J (2013). Health behavior theories in diet apps. J Consum Health Internet.

[23] Cowan LT, Van Wagenen SA, Brown BA, Hedin RJ, Seino-Stephan Y, Hall PC, West JH (2013). Apps of steel: are exercise apps providing consumers with realistic expectations?: a content analysis of exercise apps for presence of behavior change theory. Health Educ Behav.

[24] West JH, Hall PC, Hanson CL, Barnes MD, Giraud-Carrier C, Barrett J (2012). There's an app for that: content analysis of paid health and fitness apps. J Med Internet Res.

[25] Fogg BJ (2002). Persuasive Technology: Using Computers to Change What We Think and Do.

[26] Watts S, Mackenzie A, Thomas C, Griskaitis A, Mewton L, Williams A, Andrews G (2013). CBT for Depression: a pilot RCT comparing mobile phone vs. computer. BMC Psychiatry.

[27] Kenny R, Dooley B, Fitzgerald A (2015). Feasibility of “CopeSmart”: a telemental health app for adolescents. JMIR Ment Health.

